# Digital light processing of hydrogel molds to guide cell mechanosensing and the fabrication of meniscal tissue constructs

**DOI:** 10.1063/5.0289256

**Published:** 2025-11-07

**Authors:** Alysse DeFoe, Joshua Toth, Abhishek P. Dhand, Lovie Deloney, Mackenzie Obenreder, Vivek B. Shenoy, Jason A. Burdick

**Affiliations:** 1 Department of Chemical and Biological Engineering, University of Colorado Boulder, Boulder, Colorado 80303, USA; 2 Department of Materials Science and Engineering, University of Pennsylvania, Philadelphia, Pennsylvania 19104, USA; 3 Center for Engineering Mechanobiology, University of Pennsylvania, Philadelphia, Pennsylvania 19104, USA; 4 Department of Bioengineering, University of Pennsylvania, Philadelphia, Pennsylvania 19104, USA; 5 BioFrontiers Institute, University of Colorado Boulder, Boulder, Colorado 80303, USA

## Abstract

The organization of cells and extracellular matrix (ECM) informs tissue function. This structure/function relationship is especially evident in musculoskeletal (MSK) tissues in which a specific ECM organization (e.g., anisotropy) guides directional properties under load. Injury disrupts the ECM structure, and new methods are needed to recapitulate the organization of cells and ECM within MSK tissue constructs to improve tissue models as well as to fabricate implants for repair. To address this, we use digital light processing (DLP) to rapidly 3D print custom molds that support large (centimeter-scale) meniscal tissue construct formation from meniscal fibrochondrocytes embedded within collagen gels. Importantly, these hydrogel molds include multiple pillars designed to anchor the tissue constructs and provide biophysical cues to direct tissue organization. Here, the effect of pillar spacing aspect ratio, mold size, and mold curvature on tissue contraction and cellular organization is investigated both experimentally and *in silico*. Pillar placement results in either disorganized (1:1 pillar spacing) or anisotropic (1:2 or 1:4 pillar spacing) cell spreading, with anisotropy observed in molds ranging from 6 mm to 2.4 cm in length. The introduction of mold curvature does not impact final construct width but does increase cellular anisotropy relative to molds without curvature. Furthermore, culture in the presence of the contractility inhibitor Cytochalasin D reduces construct contraction. These observations of cell behavior and construct compaction based on mold design are supported by coarse-grain simulations. Overall, this work establishes an adaptable DLP-based platform to grow custom MSK constructs for tissue models or repair.

## INTRODUCTION

The organization of cells and their extracellular matrix (ECM) gives rise to tissues with specialized mechanical properties and complex functions. This relationship is especially evident in the highly organized tissues of the musculoskeletal (MSK) system, including meniscus, muscle, and cartilage.^1–3^ For example, the circumferentially aligned fibers of the meniscus allow for the conversion of high compressive loads into hoop stresses, whereas the highly aligned hierarchical components of skeletal muscle enable rapid conversion of chemical energy into directed mechanical energy and force transmission.^1,3^ Furthermore, fibers of the superficial zone of articular cartilage are aligned parallel to the articulating surface to provide a low-friction interface for joint articulation, while the fibers of the deep zone are aligned perpendicular to the surface to provide resistance to compressive load.^2^ Unfortunately, MSK tissues are frequently damaged through traumatic injury (e.g., tears and large volumetric tissue loss), disease, or degeneration, which ultimately results in the loss of tissue organization and diminished function. Thus, there is a need for methods that can organize cells and ECM within tissue constructs for use as tissue models in health and disease, as well as for use as therapeutics.

There have been great advances in biomaterial scaffold fabrication approaches (e.g., 3D printing and electrospinning) to replicate MSK tissue organization; however, many of these methods rely on specialized and complex processes and advanced material design.^4–13^ In contrast, the introduction of simple boundary constraints has been utilized as a tool to guide cell mechanosensing and introduce anisotropy to cellular behavior by leveraging signals that are found during tissue development and maturation.^14–19^ As such, a variety of tissue engineering approaches have been pursued to harness the impact of boundary conditions on cell-laden ECM gels for the reorganization of an initially isotropic matrix into a highly organized tissue construct.^20–34^ By replicating important aspects of the tissue microenvironment (e.g., cellular and fiber organization, cell–cell interactions, and cell–matrix interactions), these constructs have been especially useful as tissue models (e.g., tissue morphogenesis, cell:ECM crosstalk, cellular regulation of fiber formation, injury, wound healing, disease, and drug response) and hold promise for application as functional engineered tissue replacements.

As an early example, Legant *et al.* used small (<1 mm length) polydimethylsiloxane (PDMS) wells with pillars to form anisotropic microtissues from fibroblasts to evaluate matrix remodeling and tissue force generation over time.^24^ Expanding on this work, Bose *et al.* investigated the influence of PDMS well geometry and pillar placement on regional fibroblast-laden microtissue architecture and stiffness.^23^ In contrast to the use of pillars, Mondrinos *et al.* anchored ECM gels through chemical interactions within PDMS chambers to create anisotropic fibroblast-, mesenchymal stromal cell (MSC)-, and myoblast-sculpted constructs (∼8 mm in length) for use as microphysiological models of injury and disease.^22^ Puetzer *et al.* developed larger (∼2.5 cm gross width) anatomically shaped meniscal fibrochondrocyte (MFC)-laden constructs through mechanical anchoring of high collagen density gels over 8-week cultures to direct circumferential and radial fiber organization.^20^ Furthermore, Puetzer *et al.* demonstrated that clamping of tenocyte-, ligament fibroblast-, and MFC-laden collagen gels (1.5 × 8 × 30 mm) for 6 weeks produced highly organized collagen scaffolds with properties corresponding to the respective tissue source.^21^

These are just a few examples within this broad field where controlled matrix remodeling has been used to introduce structure into engineered tissues. Within these approaches, boundary conditions (in the form of physical or chemical anchors) are imparted by custom devices that are manufactured via manual multi-step processes (such as multilayer photolithography or injection molding) that are often tedious and/or require custom mechanical parts. The intensive manual processing, geometric limitations, and inflexible sizing of these device fabrication processes limit the rapid prototyping of devices with complex custom boundary constraints and therefore reduce the adaptability of these technologies for broad applications.

**FIG. 1.**
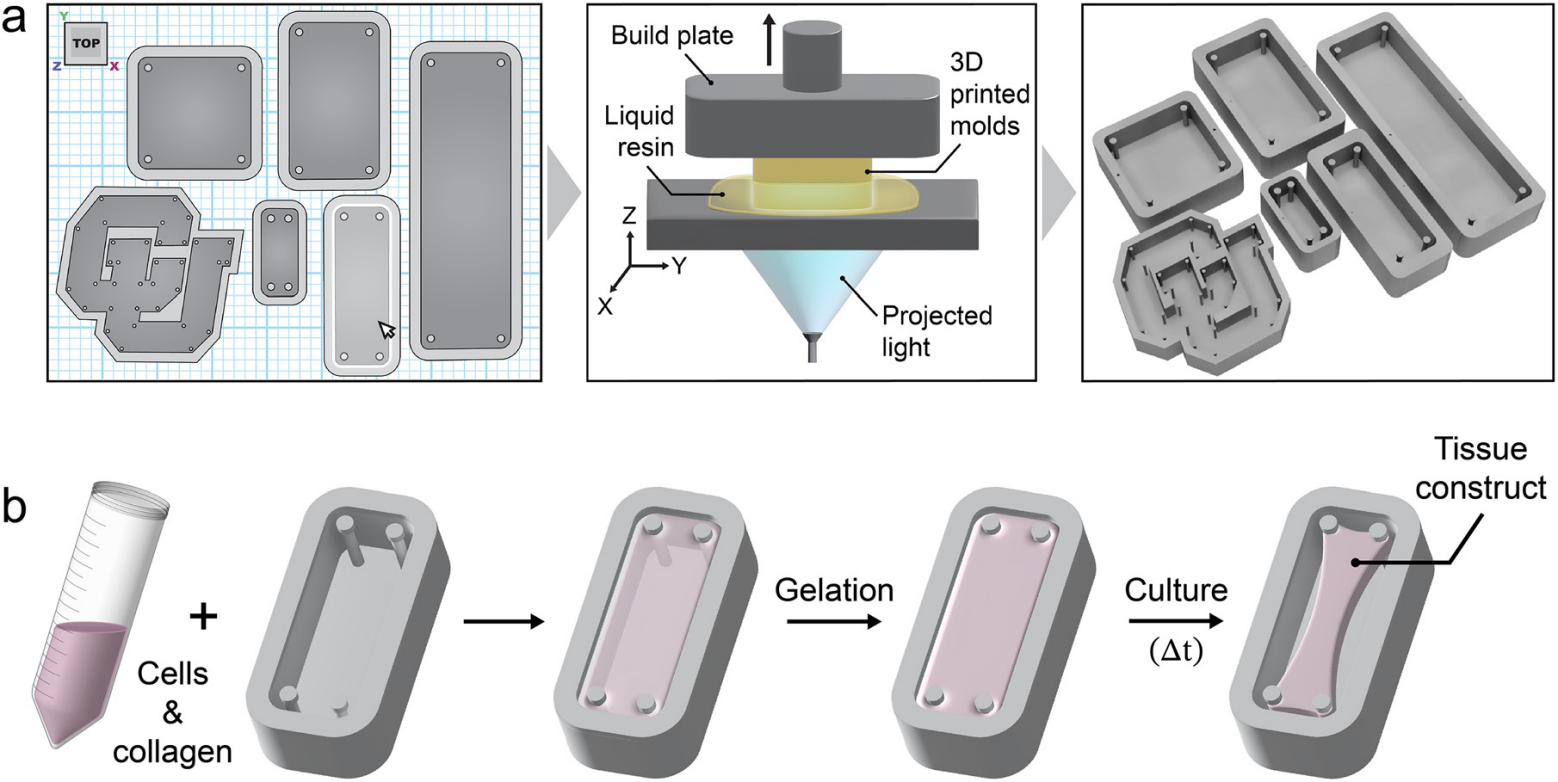
Overview of the design and fabrication of meniscal tissue constructs via DLP-printed molds. (a) Custom hydrogel molds are designed with CAD software and then printed via DLP, a process where a liquid resin is crosslinked through controlled light exposure in a layer-by-layer manner. (b) To fabricate meniscal tissue constructs, a liquid mixture of collagen and meniscal cells (i.e., MFCs) is pipetted into DLP-printed molds and placed at 37 °C to undergo gelation. Upon gelation, culture media is added. Extended culture (e.g., up to 1 week in this study) results in collagen remodeling, matrix contraction, and emergent cellular organization.

To address this need, digital light processing (DLP) has recently been adapted to rapidly fabricate reproducible microtissue devices.^35^ DLP is a 3D printing technique wherein a photoreactive resin is exposed to patterned light in a layer-by-layer manner to build up a 3D object defined by an input computer-aided design (CAD) model.^36^ Here, we utilize DLP and an inexpensive custom poly(ethylene glycol) diacrylate (PEGDA) resin for the rapid on-demand fabrication of hydrogel molds with pillars. These molds guide the formation of tissue constructs with spatial cellular organization, which is demonstrated in the context of meniscal tissue (Fig. 1). Specifically, we show that the precise control over custom-engineered boundary conditions (e.g., mold size, shape, and pillar placement) imparted by DLP printing allows for the reproducible fabrication of hydrogel molds that both facilitate and direct collagen compaction by MFCs, resulting in tissue construct formation. Furthermore, we combine experimental testing and simulations to better understand the remodeling of the ECM and change in construct shape based on these custom boundary conditions and culture environment (e.g., soluble factors that alter cell contractility). This work establishes a foundation and rapid workflow for the rational design and fabrication of pillar-based molds via DLP for directing and studying cell organization and even toward growing large custom MSK tissues for models or tissue replacement after injury.

## RESULTS

### DLP-printed hydrogel molds support the formation of complex meniscal tissue constructs

Toward the goal of accessible hydrogel mold fabrication to facilitate tissue construct formation, a simple resin was formulated from commercially available PEGDA (macromer), lithium phenyl-2,4,6-trimethylbenzoylphosphinate (LAP, photoinitiator), and tartrazine (photoabsorber) [Fig. S1(a)]. Owing to its chemical and biological inertness, PEGDA makes an ideal choice of macromer. The concentrations of photoinitiator and photoabsorber were tuned to achieve high print fidelity and resolution by controlling the initiation rate of radical polymerization and light penetration depth within the PEGDA resin. The resultant DLP-printed hydrogels exhibited a polymer content of ∼47% and a compressive modulus of 9.4±0.9 MPa, which were maintained across 14 days when stored in Dulbecco's phosphate buffered saline (DPBS) [Figs. S1(b) and S1(c)]. This structural stability of the prints ensured maintenance of desired boundary conditions throughout culture, including maintenance of mold dimensions in both xy- and z-directions [Figs. S1(d) and S1(e)].

After thorough characterization of the printed hydrogels, 12 mm molds with four pillars spaced at a traditional 4:1 aspect ratio (AR) were designed, DLP-printed, and used to engineer meniscal tissue constructs. The ECM component for the engineered constructs was selected based on the native meniscus; the primary fibrillar component of the meniscus is type I collagen (15%–25% overall wet weight and 80% dry weight in the red zone).^37,38^ Type I bovine collagen was mixed with MFCs isolated from juvenile bovine joints, pipetted into molds, gelled at 37 °C, and immersed in growth media or fixed (day 0). Volumes of MFC-laden collagen solution and the theoretical cell seeding number used for each mold type can be found in supplementary material Table 1. MFCs visibly compacted the collagen within 24 h, demonstrating the ability of these centimeter-scale DLP-printed pillar-based molds to facilitate successful MFC remodeling of the ECM gel. Constructs were imaged, and normalized construct widths were measured on days 1, 3, and 7 [Figs. S2(a) and S2(b)]. Subsets of constructs were fixed on days 1, 3, and 7 and fluorescently stained to assess cellular alignment [Figs. S2(c) and S2(d)]. To carry out statistical comparison of cellular organization across time points, overall degree of alignment was calculated and reported in the form of the alignment index (AI, value: 1 random orientation, value: 4.5 total alignment).^4,20,28^ Nuclear and actin alignment were observed by day 3 [Fig. S2(e)], and no further change in alignment was observed between days 3 and 7; thus, alignment was quantified at day 3 in subsequent studies unless otherwise noted.

**TABLE I.** List of model parameters.

Parameter	Physical meaning	Value
K	Initial collagen bulk modulus	1666.7 Pa
μ	Initial collagen shear modulus	K/2.1667
$E_{f}$	Modulus of aligned fibers in tension	8.5 $\left(K+\frac{4}{3}\mu \right)$
$\lambda_{c}$	Critical stretch parameter beyond which fiber alignment is observed	1.1
$\lambda_{t}$	Transition zone width	$(\lambda_{c}-1)/4$
$\lambda_{1}$	Transition zone lower bound	$\lambda_{c}-\lambda_{t}/2$
$\lambda_{2}$	Transition zone upper bound	$\lambda_{c}+\lambda_{t}/2$
m	Strain-stiffening exponent	10
n	Transition zone exponent	5
β	Chemical “stiffness”	2.77 × 10^−3^
α	Strength of mechanosensitive feedback	2.33 × 10^−3^
$\rho_{0}$	Initial (quiescent) cell contractility	800 Pa

**FIG. 2.**
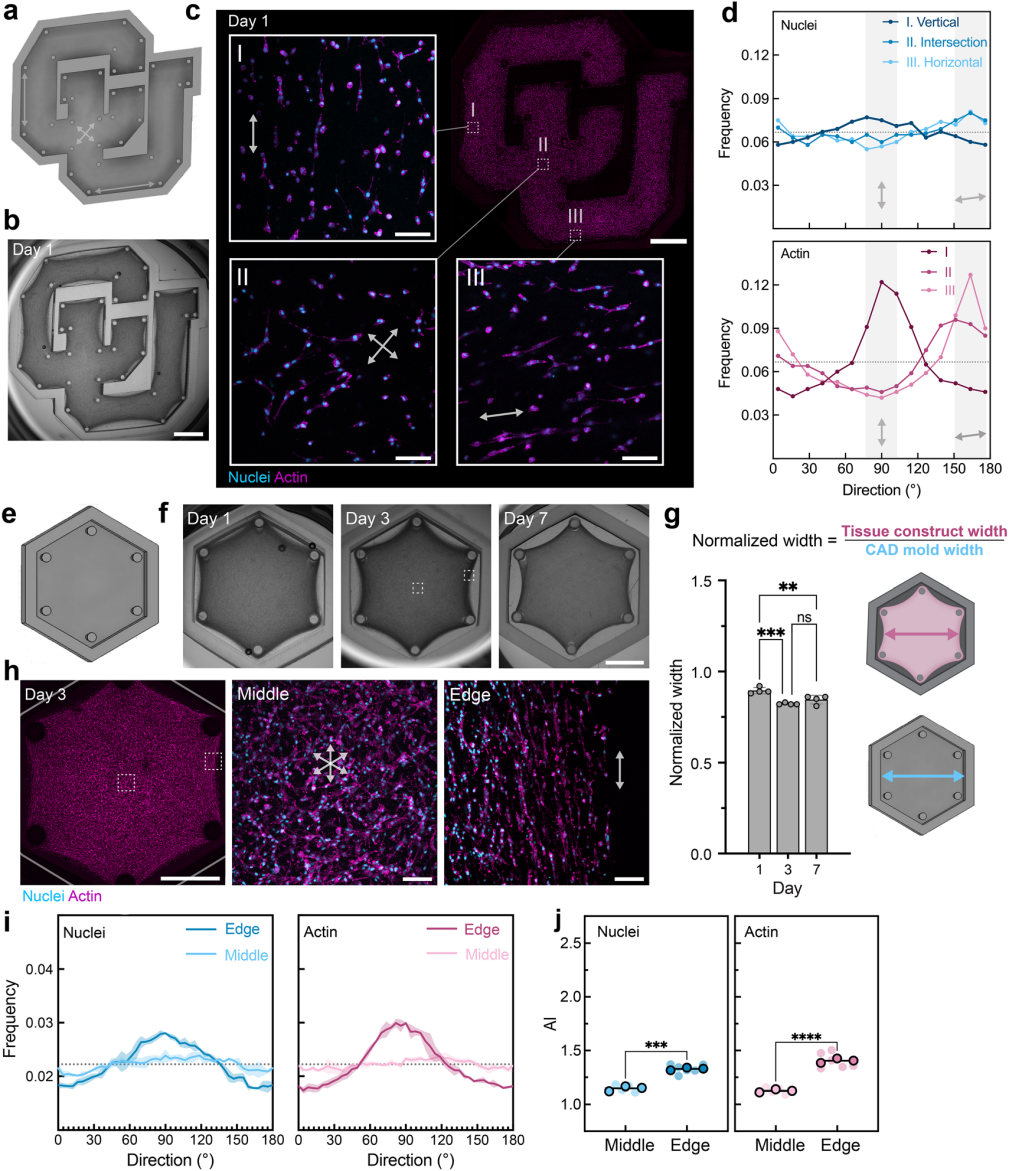
Formation of meniscal tissue constructs with complex shapes and spatially controlled cellular organization enabled by DLP-printed molds. (a) CAD model of the CU logo with 34 vertical faces and 34 pillars (arrows indicate directionality between pillars in selected regions of interest). (b) Image of DLP-printed CU logo mold and tissue construct after 1 day of culture. Scale bar: 2.5 mm. (c) Representative macroscopic fluorescence image of CU logo tissue construct (scale bar: 2.5 mm) and magnifications of three regions (I, II, III; scale bar: 50 *μ*m) at day 1, demonstrating spatially directed cellular organization [nuclei (cyan) and F-actin (magenta)]. Insets: Arrows indicate relative direction to nearest pillars. (d) Frequency distributions of nuclei and actin orientation from images I, II, and III in panel “c” as a function of direction (n = 1). Dotted gray lines indicate random cell orientation, and shaded regions denote bins that represent expected dominant directions of alignment for regions I and III based on pillar spacing. (e) CAD model of isometric hexagonal mold with six vertices and six pillars. (f) Images (dashed boxes in day 3 image indicate regions for subsequent imaging) and (g) quantification of the width of hexagonal tissue constructs normalized to mold width showing compaction across days 1, 3, and 7. Scale bar: 2.5 mm. Data are mean ± SD, n = 4 tissues, ^**^p < 0.01, ^***^p < 0.001, one-way ANOVA followed by Tukey's *post hoc* test. (h) Representative fluorescence images of cells [nuclei (cyan) and F-actin (magenta)] within the hexagonal tissue construct at day 3. Dashed boxes outline approximate locations of middle and edge images. Arrows within middle and edge images indicate relative direction to the nearest pillars. Scale bars: 2.5 mm (left); 50 *μ*m (middle and right). (i) Frequency distributions of nuclei and actin orientation as a function of direction at day 3. Shaded regions represent standard deviation of three independent tissues. (j) Alignment index (AI) of nuclei and actin for different regions at day 3. Data are reported as the mean of three independent tissues (denoted by outlined symbols) with comparisons performed using unpaired Student's two-tailed t-test, ^***^p < 0.001; ^****^p < 0.0001. Semi-transparent symbols represent the AI of individual regions (two per tissue construct).

Importantly, the versatility of DLP printing enables control over pattern dimensions, including rapid adaptation to new and intricate designs through alterations to the CAD file. To illustrate this, we first printed a mold in the shape of the “CU” logo with pillars spaced throughout and then spatially monitored collagen contraction and cellular behavior [Figs. 2(a)–2(d)]. Within 1 day, MFCs visibly contracted the collagen within the mold [Fig. 2(b)] and variations in MFC nuclei and actin organization were observed across three selected regions of interest [Fig. 2(c)]. Notably, variations in cellular orientation across these three regions corresponded to each region's position relative to nearby pillars. To illustrate this, frequency distributions of nuclei and actin orientation for these three regions are provided with select directions of the nearest pillars highlighted [Fig. 2(d)]. Peaks in these frequency distribution plots indicate higher fractions of cells oriented in the associated direction relative to other directions. As another example of spatial cellular organization, hexagonal molds with pillars around the periphery were printed and shown to support collagen compaction over time [Figs. 2(e)–2(g)]. MFCs exhibited differential alignment of nuclei and actin at the “edge” of the constructs, where the nearest pillars provided a primary principal stress axis that guided alignment in contrast to at the “middle” of the constructs, where relative pillar spacing was isotropic and lack of tension resulted in random alignment [Figs. 2(h)–2(j)].

### Mold geometry and size impact tissue construct compaction and cellular alignment

**FIG. 3.**
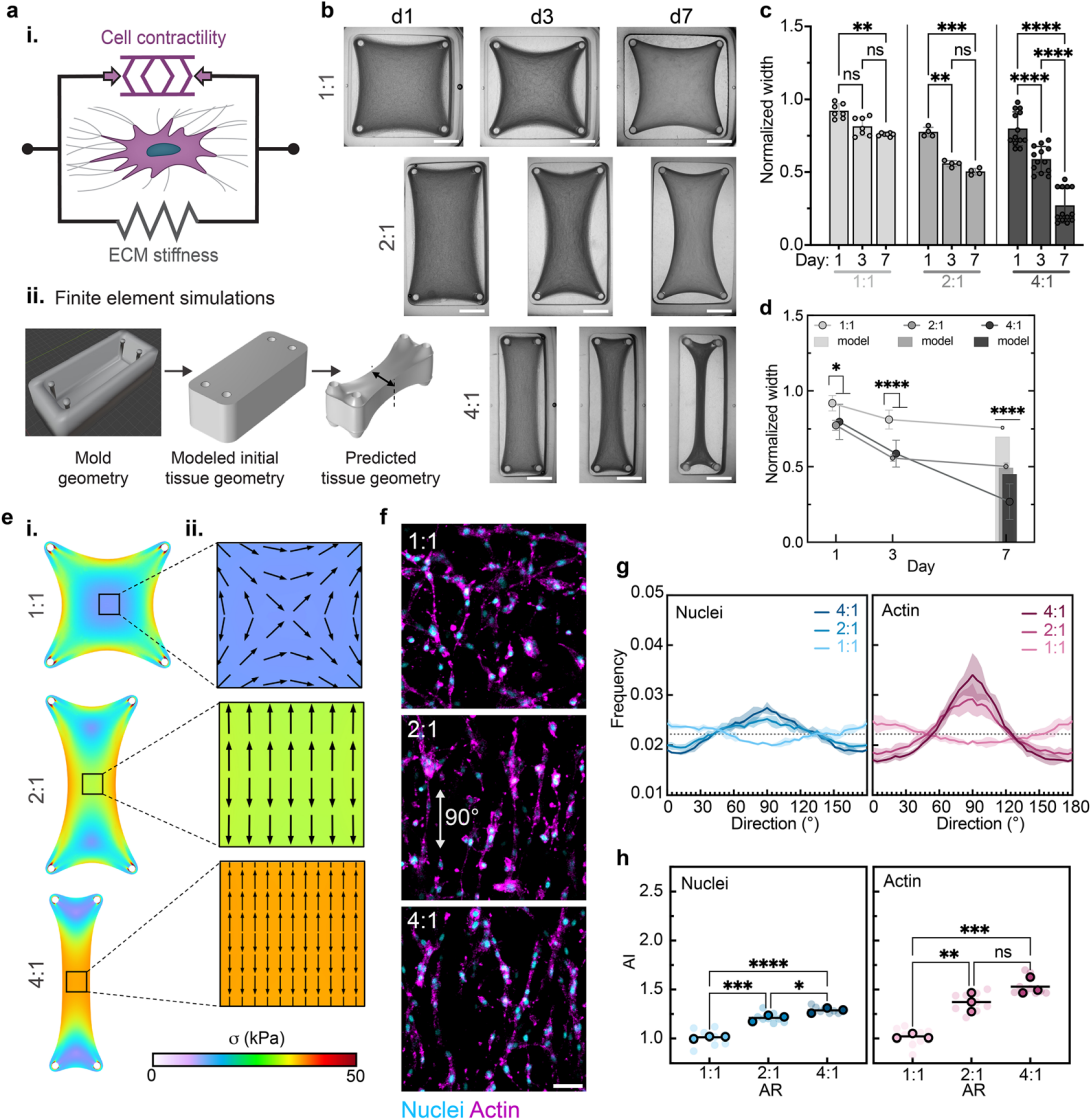
Varying aspect ratio of hydrogel mold geometries influences tissue construct shape change and cellular organization. (a) Overview of contractile tissue construct modeling and simulations. (i) Schematic of two-component coarse-grain model for contractile tissue constructs, which assumes that unaligned fibers behave as an isotropic neo-Hookean material with fibers that stiffen when aligned under tension. (ii) For each condition, the experimental mold geometry is used to model the initial tissue construct geometry upon which finite element simulations are performed to predict tissue construct geometry. (b) Images of tissue construct compaction across days 1, 3, and 7 within molds of varying aspect ratios (AR: 1:1, 2:1, and 4:1). Scale bars: 2.5 mm. Quantification of normalized tissue construct width with comparisons across (c) time and (d) AR. Data are mean ± SD, n ≥ 4 tissues, ^*^p < 0.05, ^**^p < 0.01, ^***^p < 0.001, ^****^p < 0.0001, two-way ANOVA followed by Tukey's *post hoc* test. Bars in “d” represent simulated day 7 widths. (e, i) Heat maps of simulated max principal stress across tissue constructs. (ii) Quiver plots of predicted cellular alignment and relative cellular density in the center of tissue constructs at day 7 based on simulated max principal stress direction and tissue compaction. (f) Representative fluorescence images of MFCs [nuclei (cyan) and F-actin (magenta)] at the center of tissue constructs at day 3. Scale bar: 50 *μ*m. (g) Frequency distributions of nuclei and actin orientation as a function of direction at day 3. Shaded regions represent standard deviation of three independent tissues. (h) Alignment index (AI) of nuclei and actin orientation at day 3. Data are reported as the mean of three independent tissues (denoted by outlined symbols) with comparisons performed using one-way ANOVA followed by Tukey's *post hoc* test, ^*^p < 0.05, ^**^p < 0.01, ^***^p < 0.001, ^****^p < 0.0001. Semi-transparent symbols represent the AI of individual regions (three per tissue construct).

In addition to the ability of DLP to produce complex molds with ease, DLP printing also facilitates the rapid iterative design and fabrication of unique molds with controlled dimensions. We leveraged this capacity to assess the impact of geometric mold design parameters (i.e., aspect ratio of pillar placement, mold size, and mold curvature) on overall tissue geometry and cellular organization. In parallel with experiments, we also performed simulations of the tissue remodeling process [Fig. 3(a)] to gain a better understanding of the physical mechanisms that contribute to the final tissue construct shapes and cell alignment resulting from varied initial geometries. Using a coarse-grain approach, we modeled tissue constructs as a continuum of representative volume elements that capture the presence of contractile cells actively imparting stresses on the surrounding ECM, which then passively deforms in response (Fig. S3). The collagen ECM is treated as a nonlinear fibrous material that exhibits strain-stiffening behavior arising from fiber alignment under tension, while cell contractility is treated as an initially isotropic stress applied at all points in the tissue (see Methods for a detailed explanation of model implementation). We utilized finite element simulations to solve the resulting constitutive relation [Eq. (6)] and determine the steady-state deformation and volume compaction of different tissue constructs, which are assumed to be predictive of day 7 experimental measurements. The DLP-printed mold geometries investigated were used to accurately reproduce initial tissue geometries *in silico* [Fig. 3(a)].

As a first variable, the aspect ratio (AR) of the well and pillar spacing within the hydrogel mold was varied. After one-step mold fabrication via DLP, cultures were carried out for 1 week, and compaction was observed visually [Fig. 3(b)] and quantified as construct width (normalized with respect to CAD mold widths) over time [Figs. 3(c) and 3(d)]. In particular, the low AR condition (1:1) displayed the least amount of tissue construct compaction, with a significant width change observed only between days 1 and 7. The 2:1 AR constructs underwent significant early compaction across days 1 and 3, but compaction plateaued between days 3 and 7, as indicated by no statistical difference in normalized widths between days 3 and 7. The highest AR condition (4:1) underwent the greatest compaction, with significant compaction occurring throughout the first 7 days of culture. Notably, these observations cannot solely be due to differences in initial construct volume (supplementary material Table 1), as the 2:1 constructs started with the greatest volume (315 *μ*l). While the 2:1 constructs had a greater volume than the 4:1 constructs (190 μl), we saw no significant difference between the 2:1 and 4:1 normalized widths on day 3. However, we did see very significant differences (p < 0.0001) between 1:1 (intermediate volume) and both 2:1 and 4:1 on day 3. This leads us to believe that the observed tissue shrinkage in the horizontal direction was influenced more heavily by geometric constraints than by the volume of the tissue itself. Additionally, if volume variations were more impactful than geometric constraints, we would expect to see the 1:1 and 2:1 widths continue to decrease instead of plateau between days 1 and 3 (1:1) and 3 and 7 (1:1 and 2:1). The trends here of 1:1 plateauing sooner than 2:1 and 4:1 not reaching a plateau within 7 days lead us to believe that aspect ratio is more important than initial construct volume with regard to influencing construct width over time.

When comparing across molds, the normalized widths of 2:1 and 4:1 constructs are not different at day 3, but the normalized 2:1 construct width is much greater than the normalized 4:1 construct width (^****^p < 0.0001) on day 7 [Fig. 3(d)]. This finding highlights the nonlinear relationship between AR and construct compaction over time. Although the width values did not match, simulations found similar trends in final construct widths as shown in the predicted day 7 widths and the relative shapes of representative principal stress heat maps [Figs. 3(d) and 3(e(i))].

Using simulations, cells located at the central region of constructs were predicted to show a greater degree of alignment in the elongated direction on day 7 due to stress alignment as the AR increases from 1:1 to 4:1 [Fig. 3(e)]. Note that a greater arrow density within the simulated quiver plots indicates a greater predicted cell density resulting from increased construct compaction. Experimentally, constructs cultured in isometric 1:1 molds displayed randomly aligned cells both visually and in frequency distribution plots of nuclei and actin alignment (∼0.2 regardless of direction) and when quantified via AI (∼1.0) as expected [Figs. 3(f)–3(h)]. There was no difference observed in actin alignment between 2:1 and 4:1 constructs and only a modest difference in nuclear alignment at day 3. This work indicates that some anisotropy in pillar design is needed to induce cell alignment. Thus, this implicates pillar spacing as a design parameter to control cells within tissue constructs, which is supported by prior studies assessing fibroblast-laden collagen gels in micropillar molds fabricated by alternate methods.^23,24^

**FIG. 4.**
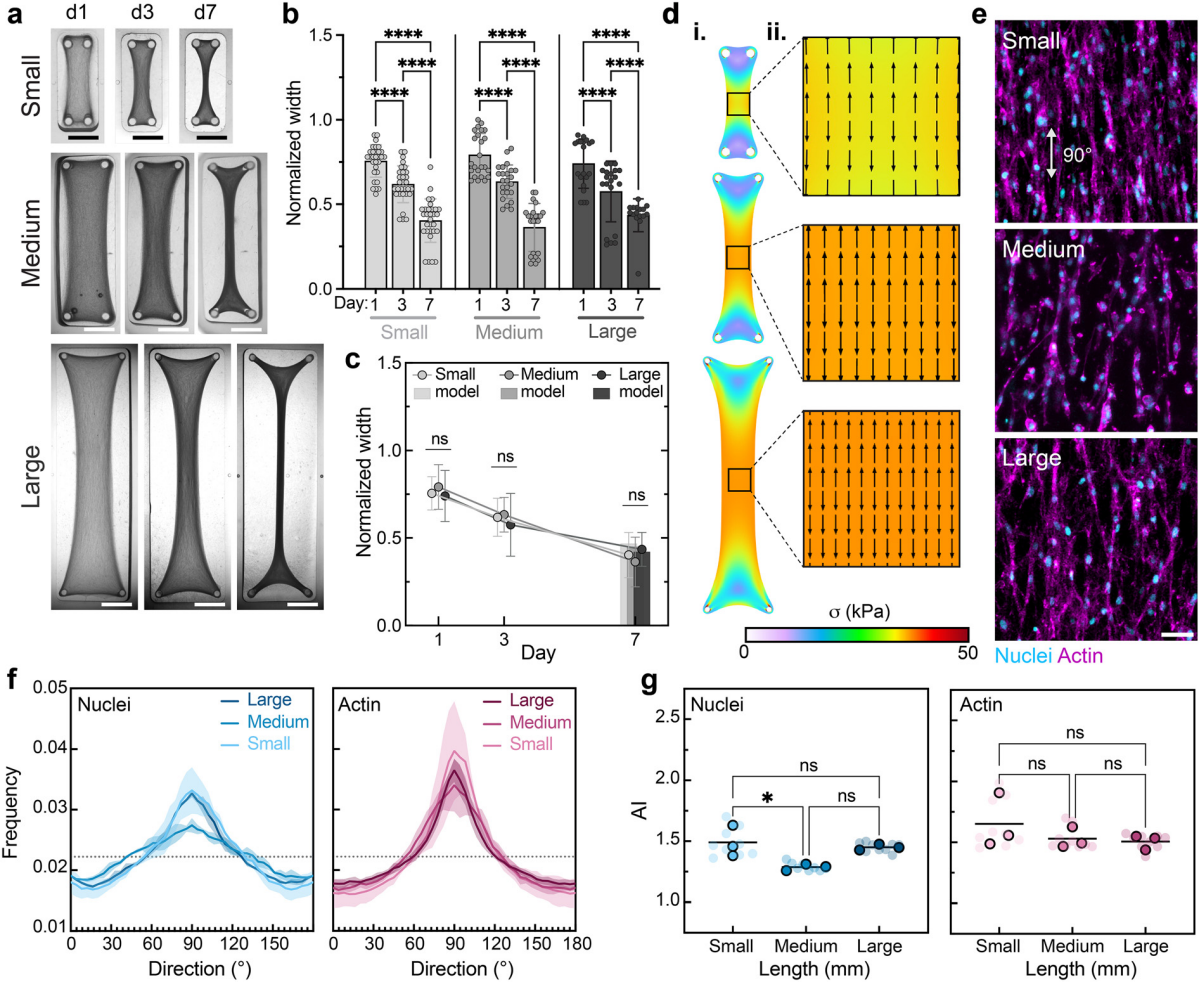
Scale of rectangular hydrogel mold geometries has little impact on tissue construct shape change and cellular organization. (a) Images of tissue construct compaction across days 1, 3, and 7 within molds of varying sizes (small: 6 mm, medium: 12 mm, large: 24 mm). Scale bars: 2.5 mm. Quantification of tissue construct width normalized to mold width with comparisons (b) across time and (c) between groups. Data are mean ± SD, n ≥ 4 tissues, ^*^p < 0.05, ^**^p < 0.01, ^***^p < 0.001, ^****^p < 0.0001, two-way ANOVA followed by Tukey's *post hoc* test. Bars in “c” represent predicted day 7 widths. (d, i) Heat maps of simulated max principal stress across tissue constructs. (ii) Quiver plots of predicted cellular alignment and relative cellular density in the center of tissue constructs at day 7 based on simulated max principal stress direction and tissue compaction. (e) Representative fluorescence images of MFCs [nuclei (cyan) and F-actin (magenta)] at the center of tissue constructs at day 3. Scale bar: 50 *μ*m. (f) Frequency distributions of nuclei and actin orientation as a function of direction at day 3. Shaded regions represent standard deviation of three independent tissues. (g) Alignment index (AI) of nuclei and actin orientation at day 3. Data are reported as the mean of three independent tissues (denoted by outlined symbols) with comparisons performed using one-way ANOVA followed by Tukey's *post hoc* test, ^*^p < 0.05, ^**^p < 0.01, ^***^p < 0.001, ^****^p < 0.0001. Semi-transparent symbols represent the AI of individual regions (three per tissue construct).

Toward the development of large aligned fibrous tissues that better mimic the dimensions of native tissues for modeling or therapeutics, we next investigated the impact of construct scale on tissue construct formation and organization. To test this, we scaled the xy pillar distances of the basic rectangular mold design (12 mm) by 50% and 200% and printed small, medium, and large molds with pillars spaced 6, 12, and 24 mm apart lengthwise, respectively [Fig. 4(a)]. As expected, we found that constructs of all scales compacted over time [Fig. 4(b)]. Interestingly, we found that the normalized construct width was independent of scale across all time points [Fig. 4(c)]. Our model also predicts that unlike in constructs of varying AR, the maximum principal stress direction and relative degree of tissue compaction at the center of constructs of varying scale are conserved, leading to minimal differences in predicted cell alignment [Fig. 4(d)]. In accordance with the model findings, other than a small change in nuclear alignment between small and medium tissues (p = 0.0407), experimental measures reveal that cellular alignment in the central region of the tissue construct is largely agnostic to the normalized scale of the tissue constructs [Figs. 4(e)–4(g)].

To investigate the impact of scale on cellular organization outside of the central region of the tissue constructs, we also imaged regions around mold posts and assessed the degree of alignment in regions at the edges of the constructs and in the intermediate region between the posts and the middle of the constructs (Fig. S4). We found that nuclear and actin alignment remained similar across the middle and intermediate regions with the exception of a decrease in nuclear alignment in the intermediate region of large tissues and a decrease in actin alignment in the intermediate region of small tissues. As expected, we observed significantly less alignment between the pairs of posts at the edges of 4:1 tissues with respect to the overall max principal stress axis. Organization in these regions changes direction, reflecting differences in stresses experienced by the cells in closer proximity to the posts. Taken together, these somewhat similar patterns in spatial cellular alignment across tissue construct scale support the conclusion that trends in cellular organization are largely agnostic to construct size and that patterns in cellular organization along the lengthwise axis of symmetry are somewhat similar across these size scales for the given collagen and cell seeding densities.

**FIG. 5.**
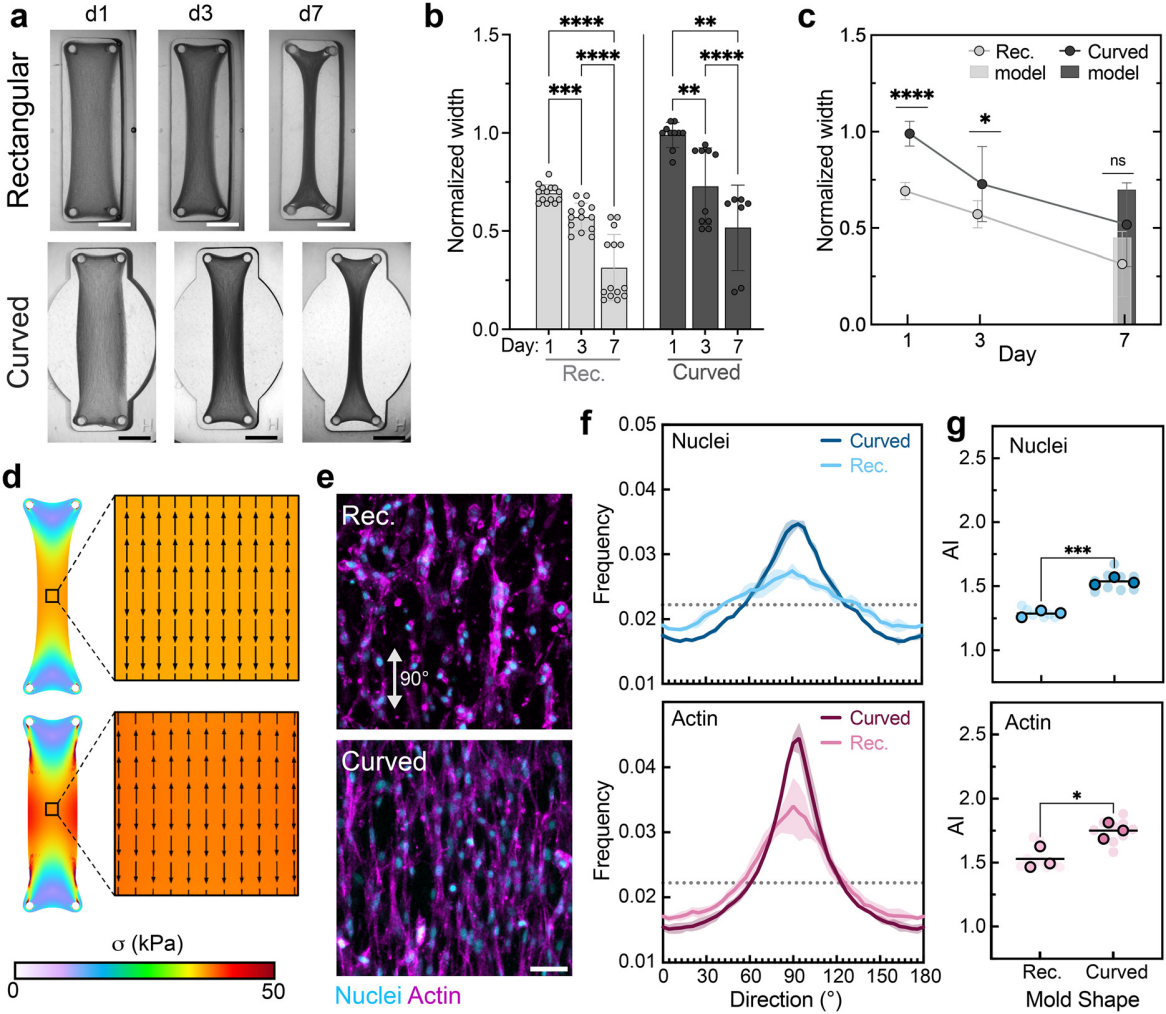
Hydrogel mold curvature has some impact on tissue construct shape change and cellular organization. (a) Images of tissue construct compaction across days 1, 3, and 7 within molds with or without curvature. Scale bars: 2.5 mm. Quantification of tissue construct width normalized to mold width with comparisons (b) across time and (c) between mold groups. Data are mean ± SD, n ≥ 4 tissues, two-way ANOVA followed by Tukey's *post hoc* test, ^*^p < 0.05, ^**^p < 0.01, ^***^p < 0.001, ^****^p < 0.0001. Bars in “c” represent predicted day 7 widths. (d, i) Heat maps of simulated max principal stress across tissue constructs. (ii) Quiver plots of predicted cellular alignment and relative cellular density in the center of tissue constructs at day 7 based on simulated max principal stress direction and tissue compaction. (e) Representative fluorescence images of MFCs [nuclei (cyan) and F-actin (magenta)] at the center of tissue constructs at day 3. Scale bar: 50 *μ*m. (f) Frequency distributions of nuclei and actin orientation as a function of direction at day 3. Shaded regions represent standard deviation of three independent tissues. (g) Alignment index (AI) of nuclei and actin orientation at day 3. Data are reported as the mean of three independent tissues (denoted by outlined symbols) with comparisons performed using unpaired Student's two-tailed t-test, ^*^p < 0.05, ^**^p < 0.01, ^***^p < 0.001, ^****^p < 0.0001. Semi-transparent symbols represent the AI of individual regions (three per tissue construct).

As a last geometric parameter, we investigated the impact of introducing mold curvature into the mold design. Limiting the tissue construct contraction that occurs over time while maintaining cellular alignment may be useful for some applications (e.g., long-term tissue construct cultures for drug testing, mechanical testing, and implantation); thus, we investigated the impact of mold curvature, a parameter that can easily be varied within printed hydrogel molds, on tissue construct shape and organization. Mondrinos *et al.* previously demonstrated that using an initial circular geometry while maintaining the same mechanical boundary conditions could prevent necking of tethered tissue constructs in the central region and prevent construct rupture.^22^ Thus, toward the goal of growing large, stable MSK tissue constructs, we examined the impact of adding mold curvature $\left(R=\frac{2}{\text{Pillar}\;\text{spacing}\;\text{length}}=\frac{1}{6}\;{\text{mm}}^{-1}\right)$ on construct shape and alignment (Fig. 5). Although we selected just one design to highlight here (plus one additional design with intermediate curvature shown in Fig. S5), the versatility of DLP could again be used to investigate a wider parameter space.

Experimentally, we found that while curved constructs initially maintained a greater normalized width than rectangular constructs, there was no significant difference in construct width by day 7 [Figs. 5(a)–5(c)]. Likewise, model results showed that despite the much greater initial width in the curved constructs, the presence of more cells exerting contractile forces in these curved tissues resulted in modest differences in predicted final widths [Fig. 5(c)] but a greater principal stress at the center of the tissue construct [Fig. 5(d)]. The addition of curvature did significantly increase the alignment of cells within the curved tissue constructs at day 3 [Figs. 5(e)–5(g)].

We speculate that this is due in part to the increased cell density in the remodeled curved constructs relative to remodeled rectangular constructs resulting from the greater number of cells seeded in the larger-volume curved constructs (cell seeding density held constant across mold type). In particular, we suggest that this increased cell density enabled the development of higher contractile stresses (as shown in simulations), which in turn facilitated increased cell alignment. It is worth noting that while increasing cell density in the rectangular mold may have given similarly superior alignment as increasing compacted cell density by incorporating curvature, those constructs would likely suffer from increased necking and risk of rupture. However, increased cell density does not explain why we observe no significant difference in alignment between moderately curved and curved constructs [Fig. S5(d)], despite differences in initial cell seeding number (curved: 135k, moderately curved: 120k). Instead, the reason why curvature increases cellular alignment in the central region of tissue constructs must extend beyond increasing cell density. For example, other factors such as altering surface area or surface stresses, changes in cell contractility, or other tissue remodeling signals may also influence tissue construct organization in the context of curved molds.

Qualitatively, compacted curved constructs appear to be denser than compacted rectangular constructs at day 7, indicating that additional curvature may also increase overall construct density while increasing cellular organization. Simulations support this finding and reveal that while the direction of max principal stress is the same at the center of remodeled rectangular and curved constructs, curved constructs undergo an overall greater degree of compaction (based on initial and final widths) and exhibit some changes in stresses at the construct edges.

### Biochemical cues influence tissue construct compaction and cellular organization

In addition to altering boundary conditions to direct tissue compaction and organization, we investigated the impact of biochemical cues on tissues cultured in DLP-printed molds. First, we increased cellular contractility through the addition of chondrogenic media containing transforming growth factor-β3 (TGF-β3). Cell viability within tissue constructs in the presence of TGF-β3 was confirmed (Fig. S6). Tissue constructs were seeded on day 0 and cultured for 24 h in growth media before switching to chondrogenic media on day 1 [Fig. S7(a)]. Early tissue compaction increased in the presence of chondrogenic media, as seen by decreased widths at day 3 relative to tissues cultured in growth media only [Figs. S7(b)–S7(d)]. However, construct widths were not significantly different at day 7, indicating that long-term compaction is not changed by increased cellular contractility [Fig. S7(d)]. In contrast to the insignificant differences in day 7 widths, cellular alignment at day 7 was greatly increased by culture in chondrogenic media relative to the growth media only condition [Figs. S7(e)–S7(g)]. As there was no difference in day 7 cell viability within tissues cultured in growth media or chondrogenic media, our findings indicate that the use of chondrogenic media in meniscal tissue culture may improve cellular organization without impeding long-term tissue geometry due to enhanced necking.

**FIG. 6.**
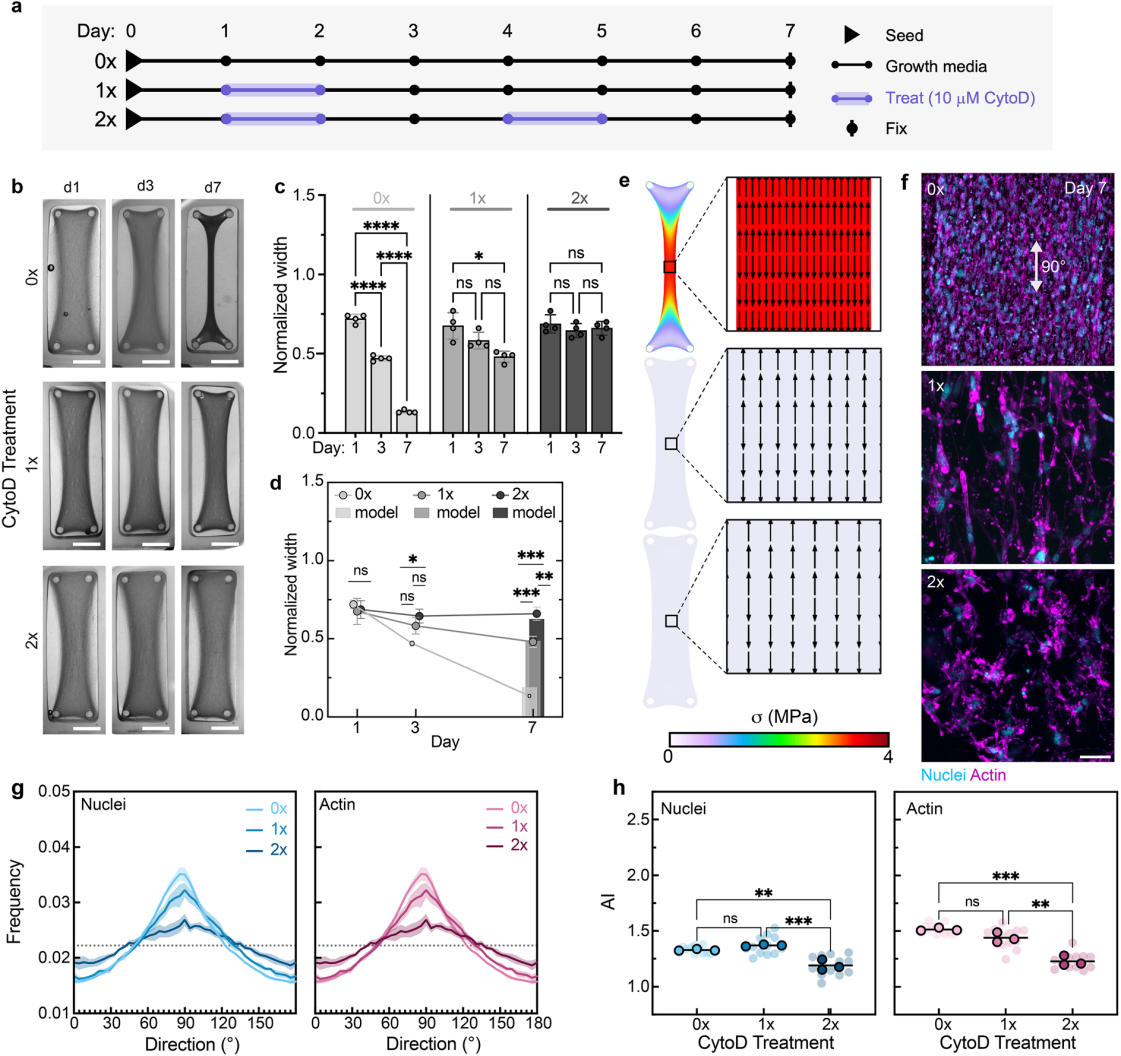
Contractility inhibitor CytoD treatment decreases macroscopic tissue construct shape change, and repeated addition of CytoD decreases cellular alignment by disrupting actin network dynamics. (a) Construct culture timelines. (b) Images of rectangular tissue constructs depicting tissue compaction across days 1, 3, and 7 with and without CytoD treatment. Scale bars: 2.5 mm. Quantification of tissue construct widths normalized to mold width with comparisons (c) across time and (d) across treatment groups. Data are mean ± SD, n ≥ 4 tissues, two-way ANOVA followed by Tukey's *post hoc* test, ^*^p < 0.05, ^**^p < 0.01, ^***^p < 0.001, ^****^p < 0.0001. Bars in “d” represent predicted day 7 widths. (e, i) Heat maps of simulated max principal stress across tissue constructs. (ii) Quiver plots of predicted cellular alignment and relative cellular density in the center of tissue constructs at day 7 based on simulated max principal stress direction and tissue compaction. (f) Representative fluorescence images of MFCs [nuclei (cyan) and F-actin (magenta)] at the center of tissue constructs at day 7. Scale bar: 50 *μ*m. (g) Frequency distributions of nuclei and actin orientation as a function of direction at day 7. Shaded regions represent standard deviation of three independent tissues. (h) Alignment index (AI) of nuclei and actin orientation at day 7. Data are reported as the mean of three independent tissues (denoted by outlined symbols) with comparisons performed using one-way ANOVA followed by Tukey's *post hoc* test, ^*^p < 0.05, ^**^p < 0.01, ^***^p < 0.001, ^****^p < 0.0001. Semi-transparent symbols represent the AI of individual regions (three per tissue construct).

We also investigated the influence of the contractility inhibitor Cytochalasin D (CytoD) on tissue compaction and organization (Fig. 6). CytoD inhibits cellular contractility by disrupting actin polymerization and depolymerization. Here, we first confirmed that CytoD did not impact day 7 cell viability within tissue constructs (Fig. S8). Then, constructs were seeded on day 0, and CytoD treatment (10 *μ*M for 24 h) was administered on day 1 or days 1 and 4. Constructs treated with a single dose of CytoD exhibited decreased tissue compaction as evidenced by insignificant differences in widths across days 1 and 3 and days 3 and 7, with a slight decrease in width across days 1 and 7 [Fig. 6(c)]. Meanwhile, constructs treated with two doses of CytoD exhibited no tissue compaction across the selected time points [Fig. 6(c)]. Portions of these constructs remain in contact with the edges of the molds due to the limited overall construct compaction resulting from reduced cell-generated contractility in the presence of CytoD [Fig. 6(b)].

At day 7, there were significant differences in tissue widths across all three groups (no CytoD, one dose of CytoD, and two doses of CytoD), suggesting a treatment-frequency-dependent response on tissue compaction [Fig. 6(d)]. These differences in tissue width were also reflected in the simulated tissue shapes [Fig. 6(e)]. Due to decreased levels of compaction, CytoD-treated constructs were predicted to have lower cell densities at day 7, as reflected by the lower arrow densities in the associated quiver plots; this decreased cell density was also observed experimentally via confocal microscopy [Fig. 6(f)]. Interestingly, constructs treated with CytoD only on day 1 retained day 7 cellular organization, with no difference in alignment index relative to the untreated control [Figs. 6(g) and 6(h)]. However, constructs treated with CytoD on days 1 and 4 exhibited significantly decreased day 7 cellular organization [Fig. 6(h)]. This finding indicates a nonlinear relationship between tissue compaction and cellular alignment in which tissue compaction can be somewhat tempered via contractility inhibitor treatment without also tempering cellular alignment during 1 week of culture.

## DISCUSSION AND CONCLUSION

In this work, DLP printing was used to fabricate hydrogel molds that impart control over engineered construct boundary conditions, thereby guiding cell mechanosensing and the repeatable formation of tissue constructs with anisotropic cell organization. DLP printing supports the printing of feature sizes down to tens of micrometers, which allows the representation of many tissue types and local heterogeneities that are observed in healthy and diseased tissues. DLP printing also allows for simple and rapid alterations in mold design, an advance over traditional approaches to fabricating such molds. Furthermore, DLP printing is a relatively low-cost method, as there are a wide range of printers that could be used for this approach. This and the use of custom resins provide accessibility in this method to many investigators.

We selected MFCs as the cell type in this study due to their ability to rapidly remodel surrounding collagen and due to similarities between this contractile tissue remodeling system and the environment of the developing human meniscus. In particular, the early meniscus consists of unorganized condensed mesenchyme with the emergence of signature aligned organization (∼13 weeks) preceded by the formation of attachments to the tibial plateau (∼10 weeks).^15,20^ Similarly, our system begins as unorganized cells and collagen, and the boundary conditions guide subsequent organization. Although we used meniscal tissue as an exemplary system, it is worth noting that we believe this DLP-printed mold-based platform is amenable to many musculoskeletal cell and tissue types, where parameters such as the rate and extent of compaction will be dependent on the cell type used. Furthermore, although this study utilized collagen, a wide range of matrix molecules or a decellularized matrix could be used in future studies depending on the tissue and cell type of interest and even to model various features of disease. The matrix utilized would influence features of the system as well, including the rate of compaction observed or the signaling to encapsulated cells.

Our investigation of the effect of geometric mold parameters (i.e., AR, scale, and curvature) on construct shape and cellular organization provides a framework for intentional tuning of cellular organization and construct size to meet different needs. In particular, ARs of 2:1 and 4:1 were shown to support cellular alignment within constructs. Toward the development of large (>1 cm) aligned fibrous tissues, cellular alignment was also achieved in 4:1 constructs of multiple centimeters in length, although it should be noted that alignment changes near the posts. Thus, we demonstrated the potential for DLP-printed pillar-based hydrogel molds to support the formation of fibrous tissue constructs of physiologically relevant sizes. Additionally, incorporating curvature improved early construct shape and cellular organization and appeared to produce denser constructs than those without initial curvature. This indicates that in addition to increasing cellular organization, additional curvature may also increase overall construct density and mechanical properties. With our continuum model for tissue mechanics, we showed that the deformation, compaction, and cell alignment of tissue constructs with different initial geometries can be predicted and that simulations agree with experimental observations. Finally, the platform developed in this work was very efficient, as we observed 100% tissue construct survival as defined by no construct rupture or lifting from posts through day 7, demonstrating the usefulness of the hydrogel selection and method for repeatably forming tissue constructs. Furthermore, pilot studies demonstrate that our platform can be used to support tissue construct formation and stability through longer periods, including at least 14 days of culture (Fig. S9).

In regard to biochemical cues, increasing cellular contractility through culture in chondrogenic media influenced macroscopic tissue compaction and increased cellular alignment without inducing undesirable construct rupture. This means that chondrogenic media can be used during the culture of tissue constructs with our platform, which will be important for the growth of ECM-rich MSK tissues in the lab. We also demonstrated that the addition of a contractility inhibitor decreases macroscopic tissue deformation, especially when added repeatedly during culture. This highlights how alterations in cellular contractility (e.g., with CytoD treatment) may be a useful tool in the context of culturing large aligned MSK tissues in which maintenance of both construct size and cellular organization is desired. Several questions remain regarding the physical mechanisms underlying some of these results, such as why final widths of constructs cultured in the presence of TGF-β3 matched those cultured without TGF-β3. This may be due to chondrogenic media promoting other processes within the remodeling tissues, such as increased ECM deposition, or some other aspect of cell behavior that we have not accounted for. For example, it has been observed that contractile cells can upregulate their contractile force output in response to increased mechanical stress only to a finite extent.^39^ Thus, cells throughout the bulk of the constructs may reach this saturation point and cannot exert more contractile forces regardless of treatment with biochemical cues that promote contractility. This expands upon earlier work introducing biochemical supplements to large constrained cell-laden ECM-based (e.g., collagen and fibrin) gels for the formation of highly aligned musculoskeletal tissues such as ligament, tendon, and skeletal muscle *in vitro*.^32–34,40^

These reported findings leverage advances in 3D printing to produce a new method to fabricate MSK tissue constructs, including meniscal tissue constructs, and explore how a variety of parameters related to boundary conditions and biochemical components influence tissue formation. However, the results are limited to relatively short-term cultures (up to 7 days) and investigation of a single ECM formulation and cellular component (MFCs); thus, there is ample opportunity to expand on these findings in the future toward the long-term culture of meniscal tissue constructs and in the investigation of alternative MSK and other tissues using this DLP-printing approach. Overall, this work provides a platform that can be used more broadly to grow a variety of MSK tissues in the lab for a wide array of tissue engineering applications.

## METHODS

### MFC isolation and culture

MFCs were isolated from juvenile bovine joints. Briefly, menisci were removed from knee joints under sterile conditions, cut into small pieces (∼3 × 3 × 3 mm^3^), and digested overnight with type II collagenase (1.5 mg ml^−1^) in amphotericin B-supplemented growth media [DMEM + GlutaMAX (Gibco), FBS (10% v/v), penicillin–streptomycin (P/S) (1% v/v), and amphotericin B (1% v/v)]. Any remaining tissue was then removed, and cells were expanded to 70%–90% confluency before passaging. Isolated MFCs were cultured in growth media [DMEM + GlutaMAX (Gibco), FBS (10% v/v), and P/S (1% v/v)] to passages 2–4 prior to seeding in tissue constructs. Media was changed every 2–3 days. All cell culture reagents were purchased from Gibco unless otherwise noted.

### DLP printing and mechanical characterization of hydrogel molds

All CAD models for disks and hydrogel molds were designed in Fusion 360 (Autodesk, USA). The resin for DLP printing of hydrogel molds consisted of poly(ethylene glycol) diacrylate (PEGDA, molecular weight = 700 Da, 65 vol. %), lithium phenyl-2,4,6-trimethylbenzoylphosphinate (LAP, 0.5 wt. %, Colorado Photopolymer Solutions), tartrazine (TTz, 2 mM), and Dulbecco's phosphate buffered saline (DPBS) [Fig. S1(a)]. DLP printing was conducted on a Lumen Alpha DLP printer (35 *μ*m xy pixel resolution, Volumetric Inc., USA) as described previously.^41^ The resin was dispensed into a polydimethylsiloxane (PDMS) vat and DLP-printed with a base layer exposed for 12 s to facilitate adhesion to the build platform and subsequent layers (100 *μ*m layer thickness) sequentially irradiated (3 s, 20 mW cm^−2^) until completion. After printing was complete, the printed molds were removed from the build platform, repeatedly rinsed in DPBS on a rocker for at least 72 h to remove any residual photoabsorber and unreacted macromer and photoinitiator, and stored in DPBS at room temperature until use.

Prints were swollen to equilibrium in DPBS for 72 h prior to characterization. The polymer content [Fig. S1(b)] of DLP-printed PEGDA hydrogels in the equilibrium swollen state at days 0, 7, and 14 was determined using the following equation:

$$v_{2}=\frac{{\bar{v}}_{2}}{{\bar{v}}_{1}\left(Q_{m}-1\right)+{\bar{v}}_{2}},$$ (1)

where $Q_{m}$ is the mass swelling ratio (equilibrium swollen hydrogel mass to lyophilized hydrogel mass) and ${\bar{v}}_{1}$ and ${\bar{v}}_{2}$ are the specific volumes of water (1 ml g^−1^) and PEGDA (0.87 ml g^−1^), respectively, as reported previously.^42^ Unconfined uniaxial compression testing (Q800 DMA, TA Instruments, force ramp = 0.75 N min^−1^) was performed on swollen DLP-printed disks (printed dimensions of 3 mm diameter, 1.5 mm thickness) to acquire stress–strain profiles of printed hydrogels at days 0, 7, and 14. Compressive moduli were then determined from the slope of the linear elastic region (10%–15% strain) of the stress–strain profiles [Fig. S1(c)]. Print resolution was assessed via stereomicroscopy, where print features were measured using ImageJ (NIH) and compared to their respective CAD dimensions [Figs. S1(d) and S1(e)].

### Fabrication and culture of tissue constructs

Immediately prior to use, hydrogel molds were sterilized in 70% ethanol for 5 min, rinsed with sterile DPBS, sterilized under a UV germicidal lamp for another 60 min, and rinsed again with sterile DPBS. Sterile DPBS was then aspirated from the hydrogel molds immediately prior to seeding to ensure that the mold well interior and pillars were dry.

Under ice-cold conditions, acid-solubilized type I bovine collagen (10.0 mg ml^−1^, Advanced BioMatrix) was mixed with FITC-conjugated type I bovine collagen (Sigma-Aldrich, C4361, ∼0.1 mg ml^−1^) to create a 4% FITC-labeled collagen stock (7.8 mg ml^−1^). The 4% FITC-labeled collagen stock was then mixed with ice-cold DPBS and neutralized with 0.1 N NaOH to achieve a final collagen precursor concentration of 1.8 mg ml^−1^. The collagen precursor solution was mixed with MFCs (5 × 10^6^ cells ml^−1^), and the resultant solution (1.6 mg ml^−1^ collagen, 500 000 cells ml^−1^) was pipetted into molds and incubated at 37 °C in a 5% CO_2_ incubator for 45 min for gelation. After gelation, the hydrogel molds containing cell-laden gels were immersed in growth media [DMEM + GlutaMAX (Gibco), FBS (10% v/v), and P/S (1% v/v)], which is defined as day 0 for experiments. With the exception of experiments where biochemical cues were added, a pipet tip was used around the edges of the mold on day 3 to ensure consistent limited adhesion between the tissue construct and hydrogel molds. This technique was not utilized in experiments with biochemical cues, as the presence of CytoD limited contractility so extensively that day 3 constructs were still soft and could have been easily damaged by this manipulation. For this reason, a pipet tip also was not used for the purpose of limiting adhesion in the control groups of the biochemical cue experiments. Media was replaced every 2–3 days unless otherwise noted.

To assess the effect of mold geometry on construct shape and organization, constructs were maintained throughout the experiment in growth media. To assess the effect of chondrogenic media on construct shape and organization, constructs were cultured in growth media for 24 h and then switched to chondrogenic media [DMEM + GlutaMAX (Gibco), P/S (1% v/v), ITS-X (1% v/v), ascorbic acid (50 *μ*g ml^−1^), NEAA (1% v/v), dexamethasone (0.1 μM), and transforming growth factor-β3 (10 ng ml^−1^)] for days 1–7. To assess the effect of Cytochalasin D (CytoD, 10 *μ*M) on construct shape and organization, constructs were cultured in growth media for 7 days and treated with CytoD either once (on day 1) or twice (on days 1 and 3).

### Stereoscope imaging, staining, and visualization of tissue constructs

Tissue constructs were imaged via stereomicroscopy on days 1, 3, and 7 to assess the overall change in construct shape with culture. Images were analyzed using ImageJ (NIH), and construct width was measured at the mid-length of the construct. Meniscal tissue constructs were also fixed at relevant time points with warm paraformaldehyde (4% v/v) for 15 min or overnight at 4 °C, permeabilized with Triton X-100 (0.1% v/v), and stained with Alexa Fluor™ 647 phalloidin (1:400) and Hoechst 33342 (1:1000) to visualize F-actin and nuclei, respectively. Z-stack fluorescence images were acquired on a confocal microscope (Nikon Eclipse Ti2 with AXR scanner) equipped with a 2× or 10× objective lens. Z-stacks for alignment analysis were acquired in the central region of the tissue unless otherwise noted. Images were analyzed (including binarization for alignment quantification) using ImageJ (NIH), and cell and nuclei alignment within constructs were quantified using the Directionality plugin in ImageJ with batch quantification automated using the MRI_Analyze_Alignment_of_Muscles_Tool from Montpellier Ressources Imagerie.^43^

To assess MFC viability in the presence of chondrogenic media and Cytochalasin D, constructs at day 7 were incubated with Hoechst 33342 (1:1000), calcein AM, and ethidium homodimer-1 at 37 °C for 30 min per manufacturer's protocol (Live/Dead™ Cytotoxicity Kit, Thermo Fisher Scientific). Fluorescence images were acquired and binarized using a custom script in ImageJ (NIH), and nuclei were isolated using the Analyze Particles feature. The number of nuclei stained with ethidium homodimer-1 relative to the total number of nuclei (stained with Hoechst) was used to estimate the proportion of viable cells within tissue constructs.

### Modeling of ECM remodeling and construct contraction

#### Fibrous collagen ECM

A coarse-grain model for tissue mechanics was developed assuming that the main contribution to tissue mechanical properties is the network of collagen ECM fibers. The collagen ECM was modeled using a nonlinear, anisotropic constitutive law for fibrous materials that attributes mechanical behavior to two families of fibers: (1) an isotropic distribution of randomly aligned fibers and (2) a subset of fibers that align along the direction of maximum principal stretch when the matrix experiences tensile stresses and produce a strain-stiffening response.^44^ As shown in Fig. 3(e), the direction of max principal stress (and max principal stretch) that develops in the bulk of each tissue construct depends on the tissue shape and the points of attachment that define the boundary conditions as the tissue contracts. In elongated constructs, max principal stretch is primarily in the elongated direction, encouraging fiber alignment in that direction. The randomly aligned fibers are treated as a neo-Hookean hyperelastic material with strain energy density of the form:

$$W^{R}=\frac{\mu }{2}\left({\bar{I}}_{1}-3\right)+\frac{K}{2}{\left(J-1\right)}^{2},$$ (2)

where μ and K are the initial shear and bulk moduli of the matrix, respectively, J=det(F) is the Jacobian of the deformation gradient tensor, and ${\bar{I}}_{1}$ is the first invariant of the deviatoric part of the right Cauchy–Green tensor. The strain energy density of the aligned fibers can be written as

$$W^{A}=\sum_{a=1}^{3}f(\lambda_{a}),$$ (3)

where $\lambda_{a}$ are the principal stretches. The energy function $f(\lambda_{a})$ was chosen such that (i) the energy density contribution from the aligned fibers, $W^{A}$ vanishes below a critical value of tensile stretch, and (ii) the matrix stiffens only in the direction of tensile principal stretches when $\lambda_{a}$ is larger than the stretch critical value [Fig. S3(a)]. The following form for $f(\lambda_{a})$ characterized by the parameters $E_{f},n,m,\lambda_{1},\;\text{and}\;\lambda_{2}$ (Table I) was used

$$f(\lambda_{a})=\left\{\begin{array}{l} 0,\;\;\;\;\;\;\;\;\;{\;\;\;\;\;\;\;\;\;\;\;\;\;\;\;\;\;\;\;\;\;\;\lambda }_{a}<\lambda_{1}\\ E_{f}\frac{{\left(\frac{\lambda_{a}-\lambda_{1}}{\lambda_{2}-\lambda_{1}}\right)}^{n}{\left(\lambda_{a}-\lambda_{1}\right)}^{2}}{\left(n+1\right)\left(n+2\right)},\;\;\;\;\;\;\;\;\;\;\;\;\;\;\;\;\;\;\;\;\;{\;\lambda }_{1}\leq \lambda_{a}\leq \lambda_{2}\\ E_{f}(\frac{{\left(1+\lambda_{a}-\lambda_{1}\right)}^{m+2}-1}{\left(m+1\right)\left(m+2\right)}+\frac{\lambda_{2}-\lambda_{a}}{m+1}+\frac{\left(\lambda_{a}-\lambda_{2})(\lambda_{2}-\lambda_{1}\right)}{n+1}+\frac{{\left(\lambda_{2}-\lambda_{1}\right)}^{2}}{\left(n+1\right)\left(n+2\right)}),{\;\lambda }_{a}>\lambda_{2}. \end{array}\right.$$ (4)

The transition region $\lambda_{1}\leq \lambda_{a}\leq \lambda_{2}$ is included to ensure $f(\lambda_{a})$ is a smooth function.

#### Tissue contraction

It is assumed that upon initial creation, cells within tissue constructs are homogeneously distributed throughout the tissue volume with an unpolarized actomyosin cytoskeleton, showing no preferential alignment or exertion of contractile stresses. Therefore, in a coarse-grain approximation, myosin motors act as force dipoles and exert an isotropic applied stress, $\rho_{0}$, at every point in the tissue [Fig. S3(b)]. Experiments have shown that the magnitude of an adherent cell's contractility increases over time when placed inside a matrix, as stress generated in the matrix activates mechanosensitive signaling pathways that upregulate contractility, such as the rho–ROCK pathway or the calcium–calmodulin pathway.^45,46^ A mathematical framework was developed to describe how these processes affect a cell's contractility at steady state in previous work.^47^ The same framework is applied here to determine the increased level of contractility in tissue constructs with remodeling. Effective contractility, $\rho_{\text{eff}}$ is found as

$$\rho_{\text{eff}}=\frac{\beta \rho_{0}}{\beta -\alpha }.$$ (5)

Equation (5) describes how the initial level of cellular contractility in the absence of feedback from mechanosensitive signaling, $\rho_{0}$, is modulated by the activation of this signaling characterized by the parameters α and β. Physically, α relates the strength of the mechanosensitive pathways coupling feedback between stress and contractility and β represents the penalty for any change in contractility from its initial, quiescent value $\rho_{0}$. Values of all model parameters are listed in Table I.

#### Simulation procedure

The constitutive equation used to model tissue constructs is obtained by combining Eqs. (2)–(5)

$$\sigma_{ij}=K\left(\;J-1\right)\delta_{ij}+\left(\frac{1}{\;J}\right)\mu {\bar{B}}_{ij}+\frac{1}{J}\sum_{a=1}^{3}\frac{\partial f\left(\lambda_{a}\right)}{\partial \lambda_{a}}\lambda_{a}\left(n_{a}⊗n_{a}\right)+\frac{\beta \rho_{0}}{\beta -\alpha }\delta_{ij},$$ (6)

where $\bar{B}$ is the deviatoric part of the modified left Cauchy–Green tensor and $n_{a}$ are the unit vectors of the principal stress orientations. The steady-state stress and strain fields are obtained using the finite element method in COMSOL Multiphysics^®^ software to solve the force-balance equation $\partial \sigma_{ij}/\partial x_{i}=0$. Dimensions of the molds used to create tissues in experiments were used to create identical initial tissue geometries in the simulations. Fixed boundary conditions were applied on regions where tissues contact the mold pillars. The width of the steady-state deformed tissue geometry is reported as the predicted day 7 tissue width. A sensitivity analysis was performed to examine the impact of the initial contractility, $\rho_{0}$, and the bulk modulus, K, on predicted tissue width to determine parameter values that produced a good fit with experimental measurements from tissues of different sizes (6, 12, and 24 mm) [Fig. S3(c)]. The same parameter values were then used when modeling all other tissue constructs. To model the inhibition of tissue contractility from specific chemical treatment, the value of initial cell contractility, $\rho_{0}$, was reduced by 20% (CytoD+) or 60% (CytoD++). Cell alignment was predicted from simulation results under the assumption that cells at a given location in a tissue will preferentially align along the direction of maximum principal stress and that alignment will depend on the degree of compaction that cells experience. Accordingly, an alignment index was calculated across a 1 × 1 × 1 mm^3^ region at the center of each tissue by multiplying a volume change factor (ratio of initial to final volume) and an order parameter representing the degree of alignment of maximum principal stress within the region. The order parameter was found as the largest eigenvalue of the tensor $Q_{ij}$

$$Q_{ij}=\frac{1}{2}(3n_{i}n_{j}-\delta_{ij}),$$ (7)

where n represents the unit vector associated with the direction of maximum principal stress. Note that calculating the invariant tensor $Q_{ij}$ is a common practice for determining molecular alignment, such as in the case of a nematic liquid crystal, and we extend it to coarse-grained simulations here by calculating $Q_{ij}$ at every point within the region considered and then taking a volume average.^48^

### Statistical analysis

Comparisons of two groups were performed using an unpaired Student's two-tailed t-test. Comparisons across three or more groups with one categorical variable were performed using an ordinary one-way ANOVA, and comparisons across three or more groups with two categorical variables (such as shape, size, or treatment and time) were performed using a two-way ANOVA or a mixed-effects analysis. Comparisons across three or more groups were followed by Tukey's *post hoc* multiple comparisons test. For all analyses, ^*^p < 0.05, ^**^p < 0.01, ^***^p < 0.001, ^****^p < 0.0001. All statistical analyses were performed using GraphPad Prism version 10.

## SUPPLEMENTARY MATERIAL

See the supplementary material for additional information, including print characterization, additional tissue construct conditions, modeling information, and viability analyses.

## Data Availability

The data that support the findings of this study are available from the corresponding author upon reasonable request.
